# The Significance of Symptoms in Predicting Coronary Artery Aneurysms of Kawasaki Disease, Especially in Female Patients

**DOI:** 10.3389/fcvm.2022.823862

**Published:** 2022-04-28

**Authors:** Huan Yu, Weiyue Sun, Haoran Wu, Shuchi Zhang, Zhipeng Xu, Rongzhou Wu, Xing Rong, Huixian Qiu, Jinshun Zhu, Chunxiang Zhang, Maoping Chu

**Affiliations:** Department of Pediatrics, Second Affiliated Hospital and Yuying Children’s Hospital of Wenzhou Medical University, Wenzhou, China

**Keywords:** Kawasaki, coronary artery aneurysms, rash, oral changes, cervical lymphadenopathy, female

## Abstract

**Background:**

Kawasaki disease (KD) is an acute febrile systemic vasculitis of unknown etiology that occurs during early childhood, commonly involving the coronary artery, and can lead to coronary artery aneurysms (CAAs).

**Methods:**

The demographic, clinical, and laboratory data of KD patients without coronary artery lesions (N-CAL) and with CAA were collected during 2005–2020 at the Second Affiliated Hospital of Wenzhou Medical University. The patients were divided into the development cohort and the validation cohort. First, we compared the general information, symptoms, and laboratory data of N-CAL and CAA patients in the development cohort and the total cohort and screened out the different indices by logistic regression analysis. Then, we established three models and compared the area under the curve (AUC) values of the receiver operating characteristic (ROC) curves to identify meaningful models for CAA, which were further verified by decision curve analysis (DCA). Second, taking into account previous reports on the importance of gender to CAA, gender stratification was conducted.

**Results:**

The analysis of clinical and blood indices revealed the following novel features: (i) Many factors were found to be related to CAA, including IVIG resistance and the symptoms of rash, oral changes, and cervical lymphadenopathy. (ii) The development cohort was analyzed by logistic regression, and three models were established. The ROC curves showed that Model 2, composed of IVIG resistance, rash, oral changes, and cervical lymphadenopathy, had a better AUC value and easily to evaluate in the prediction of CAA. (iii) The selected model for predicting CAA in the development cohort was further confirmed in the validation cohort through DCAs. (iv)We further compared the items enrolled in the three models above between the N-CAL and CAA groups by sex, and the results indicated that female KD patients without rash, oral changes, and cervical lymphadenopathy were more likely to develop CAA.

**Conclusion:**

The absence of rash, oral changes, and cervical lymphadenopathy are risk factors for CAA, especially in female KD patients. Accurately recognizing symptoms, early diagnosis, and standard treatment for KD are key to reducing the incidence of CAA.

## HIGHLIGHTS

- The results are more reliable because of the large numbers of patients and the wide span of years.
- This is the first study to indicate the importance of the three symptoms (rash, oral changes, and cervical lymphadenopathy) in predicting CAA in KD patients. More importantly, this finding can be easily applied to clinical practice.
- This is the first study to demonstrate that the absence of rash, oral changes and cervical lymphadenopathy are more prominent predictors of CAA in female KD patients.

## Introduction

Kawasaki disease (KD) is an acute systemic vasculitis disease that mainly occurs in children under 5 years of age and affects small- and medium-sized blood vessels, especially the coronary arteries. Although intravenous immunoglobulin (IVIG) decreases the incidence of coronary artery lesions (CALs) from 25 to 4% (1), KD has become the leading cause of secondary heart damage in children worldwide (2–4).

Studies have shown that CAL in Kawasaki disease may persist and develop into stenosis and occlusion (5). Coronary artery aneurysms (CAAs) cause serious damage to children in particular. In recent years, IVIG resistance and incomplete Kawasaki disease (IC-KD) has been increasingly recognized by researchers and clinicians. IVIG resistance is defined as persistent fever 36–48 h after IVIG or recrudescent fever within 1 week (6). Studies suggest that IVIG resistance reflects the severity of underlying inflammation in children, which leads to an increased risk of CAA (7).

The main clinical manifestations of KD include fever, rash, oral changes, anomalies of the extremities, bilateral non-exudative conjunctivitis, and cervical lymphadenopathy. Fever with four or five clinical symptoms is considered to be complete KD. Fever with less than four clinical symptoms is considered to be incomplete KD (8). Clinical symptoms alone are insufficient, and other laboratory tests play an auxiliary role in the diagnosis of incomplete KD, including increased platelets (PLT), decreased serum albumin (ALB), low serum sodium (Na), and many other indices. Incomplete KD is also considered to be one of the risk factors for CAA (9).

Studies have shown that anemia is an independent risk factor for CAA (10). Other studies found that male sex, delayed treatment with IVIG, increased c-reactive protein (CRP) (11), extreme age (12), and tachycardia (13) were all risk factors for CAA (14, 15). Now, using the data from a children’s hospital in southeastern China over several years, we retrospectively analyzed the characteristics of CAA patients and conducted further research so that the features of CAA patients—one of the most serious complications in KD—can be understood better.

## Materials and Methods

### Participants and Study Design

Our study was approved by the Ethics Committee of the Second Affiliated Hospital and Yuying Children’s Hospital of Wenzhou Medical University. We collected data from Kawasaki disease patients who were hospitalized in the Second Affiliated Hospital and Yuying Children’s Hospital of Wenzhou Medical University from 2005 to 2020.

Admission criteria were as follows: fever for more than 5 days, accompanied by the following 4 or 5 clinical manifestations; bilateral conjunctival injection, oral changes, cervical non-suppurative lymphadenopathy, rash, and extremity changes. Patients with these symptoms were diagnosed with complete Kawasaki disease. Patients with only three or fewer concomitant symptoms were diagnosed with incomplete KD.

Exclusion criteria were as follows: (1) Not in the acute stage; (2) Not treated with IVIG; (3) Uncertain diagnoses that cannot be distinguished from other diseases, such as septicemia and juvenile idiopathic arthritis.

For the purpose of our study, to better characterize the features of CAA patients, we grouped the patients into N-CAL and CAA, and compared them. We included patients who had not developed CAL from the onset of the disease to the end of the follow-up as the N-CAL group. The patients with CAA at any stage from the onset of the disease to the end of the follow-up were classified into the CAA group. To make our results more reliable, the total patients were randomly divided into the development cohort and the validation cohort with the R package caret. A total of 2/3 were included in the development cohort, and 1/3 were included in the validation cohort. By comparing the data of the N-CAL and CAA groups in the development cohort and the total cohort, we found that CAA patients had lower rates in rash, oral changes and cervical lymphadenopathy. Besides, the three symptoms were proved to be significant in predicting CAA by establishing models, which were verified in the validation cohort. Considering that KD is more prevalent in male gender, we conducted gender stratification to further comparison. The results indicated that the absence of rash, oral changes, and cervical lymphadenopathy were better predictors of CAA in female KD patients.

### Clinical and Laboratory Data

Considering the subjectivity of the diagnosis of Kawasaki disease, the clinical symptoms evaluation and the final diagnosis of all patients were carried out by experienced pediatric cardiologists. The blood tests and the laboratory examinations after admission were conducted after fasting and prior to IVIG. All demographic data, clinical symptoms and laboratory tests were collected by trained doctors. Echocardiography was performed by senior doctors in the Ultrasound Department of the Second Affiliated Hospital of Wenzhou Medical University to determine the existence of CAA. CAA is diagnosed mainly by echocardiography. In recent years, researchers have found that this is a better method to identify CAA based on *Z* value calculation. However, because of the large span of our cases and a large number of *Z* value deletions, we used the following criteria in this study for the definition of CAA: (1) the lumen diameter of children < 5 years old is at least 3 mm or > 5 years old at least 4 mm. (2) The inner diameter of the segment is at least 1.5 times that of the adjacent segment, and (3) the lumen is obviously irregular (16).

### Follow-Up

All patients were followed up regularly after discharge, and echocardiography was performed at approximately 1, 3 and 6 months after discharge. All the patients in the group had at least one echocardiographic follow-up result; otherwise, they were regarded as lost to follow-up.

### Data Analysis

The classified variables were described by count and percentage, and the chi-squared test was used to compare two groups. Continuous variables are expressed as the mean ± standard deviation, and t tests or Mann–Whitney *U* tests were used to compare two groups. *P* < 0.05 indicates that the difference is statistically significant. Logistic regression analysis was used for multivariate analysis. The area under the curve (AUC) value under the receiver operating characteristic (ROC) curve was used to judge the effectiveness of the model, and the decision curve analysis (DCA) was used for further confirmation.

## Results

### Many Factors Were Found to Be Related to CAA in the Development Cohort and the Total Cohort

All subjects (*n* = 1749) were randomly divided into two groups using the R package caret: the development cohort (67%, *n* = 1165) and the validation cohort (33%, *n* = 584). It has been proven suitable to include 2/3–4/5 patients as the development cohort (17, 18). The baseline values of the total cohort and the development cohort are displayed in Table 1, according to whether CAA was present. In the development cohort, more male patients (*p* = 0.01) and incomplete Kawasaki disease patients (*p* = 0.004) were found in the CAA group. No significant difference in age (*p* = 0.745) was found between the two groups. In terms of clinical symptoms, rash, oral changes, and cervical lymphadenopathy were considered to be less common in the CAA group, and the *p* values were < 0.001, < 0.001, and 0.001, respectively. Regarding the therapeutic response, the rate of IVIG resistance (*p* < 0.001) was found to be higher in the CAA group. In addition, lower hemoglobin (*p* = 0.017) and serum chloride levels (*p* = 0.011), and higher platelets (*p* < 0.001), CRP (*p* = 0.012), and ESR (*p* = 0.018) were identified in the CAA group. Similar differences in the above indicators were observed in the total cohort, and the results are shown in detail in Table 1.

**TABLE 1:** Demographic and clinical characteristics.

	Development cohort			Total		
	N-CAL	CAA	*P*	N-CAL	CAA	*P*
Male	674 (61.9%)	59 (76.6%)	0.01*	1,007 (61.8%)	90 (75.6%)	0.002*
Age (month)	28.31 ± 21.65	32.55 ± 29.25	0.745	29.87 ± 22.60	31.90 ± 28.27	0.406
Fever	1,088 (100%)	77 (100%)	1	1,630 (100%)	119 (100%)	1
Rash	873 (80.2%)	46 (59.7%)	< 0.001*	1,289 (79.1%)	67 (56.3%)	< 0.001*
Conjunctivitis	1,071 (90.9%)	66 (85.7%)	0.154	1,462 (89.7%)	101 (84.9%)	0.121
Oral	1,039 (95.5%)	61 (79.2%)	< 0.001*	1,547 (94.9%)	99 (83.2%)	< 0.001*
Extremities	804 (73.9%)	52 (67.5%)	0.23	1,192 (73.1%)	83 (69.7%)	0.455
Lymphadenopathy	587 (54%)	26 (33.8%)	0.001*	883 (54.2%)	41 (34.5%)	< 0.001*
IC-KD	193 (17.9%)	25 (32.5%)	0.004*	312 (19.3%)	41 (34.5%)	< 0.001*
IVIG-R	38 (3.5%)	11 (14.3%)	< 0.001*	57 (3.5%)	19 (16%)	< 0.001*
HB	112.44 ± 10.72	110.38 ± 10.56	0.017*	112.08 ± 10.10	108.58 ± 10.69	0.001*
PLT	370.13 ± 128.13	437.67 ± 195.32	< 0.001*	370.71 ± 126.62	424.66 ± 177.55	< 0.001*
WBC	15.88 ± 5.72	16.48 ± 6.32	0.224	15.98 ± 5.70	16.44 ± 5.96	0.164
N	10.65 ± 4.94	11.49 ± 5.19	0.395	10.84 ± 4.98	11.38 ± 4.69	0.63
L	3.86 ± 2.09	3.57 ± 2.34	0.4	3.77 ± 2.03	3.40 ± 2.13	0.313
E	0.4 ± 0.45	0.49 ± 0.62	0.106	0.39 ± 0.44	0.54 ± 0.64	0.037*
HCT	0.34 ± 0.3	0.33 ± 0.33	0.056	0.34 ± 0.31	0.33 ± 0.33	0.039*
CRP	82.73 ± 61.24	108.28 ± 59.01	0.012*	84.59 ± 61.01	108.84 ± 55.11	0.021*
ESR	37.75 ± 15.04	41.55 ± 14.96	0.018*	36.82 ± 14.52	39.68 ± 14.27	0.006*
Na	135.72 ± 2.5	134.76 ± 2.24	0.02*	135.78 ± 2.51	135.28 ± 2.47	0.104
Cl	101.84 ± 3.07	100.33 ± 3.15	0.011*	101.76 ± 3.01	100.81 ± 3.00	0.041*
Ca	2.32 ± 0.13	2.28 ± 0.14	0.346	2.31 ± 0.14	2.25 ± 0.15	0.063
Mg	0.92 ± 0.87	0.89 ± 0.11	0.429	0.91 ± 0.09	0.90 ± 0.14	0.554
P	1.41 ± 0.26	1.35 ± 0.31	0.791	1.39 ± 0.26	1.40 ± 0.34	0.157
ALT	68.3 ± 90.96	76.93 ± 90.15	0.726	70.14 ± 95.41	62.75 ± 77.60	0.41
AST	52.00 ± 70.67	61.88 ± 76.97	0.516	51.82 ± 76.65	50.31 ± 64.94	0.917
ALB	37.58 ± 5.84	37.04 ± 5.2	0.612	37.74 ± 5.81	36.07 ± 5.28	0.023*
GGT	67.69 ± 86.6	87.45 ± 87.91	0.464	68.80 ± 87.38	81.15 ± 75.41	0.36
TBIL	10.21 ± 11.78	8.62 ± 9.94	0.797	9.49 ± 10.77	8.47 ± 9.45	0.834
DBIL	3.8 ± 7.26	3.67 ± 5.57	0.568	3.50 ± 6.68	3.64 ± 5.29	0.691
IBIL	6.45 ± 5.38	4.95 ± 4.59	0.106	6.03 ± 4.82	4.83 ± 4.33	0.046*

IC-KD, incomplete Kawasaki disease; IVIG-R intravenous immunoglobulin-resistance; HB hemoglobin; PLT platelets; WBC white blood cell; N neutrophils; L lymphocyte; E eosinophil; HCT hematocrit; CRP c-reactive protein; ESR erythrocyte sedimentation rate; Na sodium; Cl chlorine; Ca calcium; Mg magnesium; P phosphorus; ALT aspartate aminotransferase; AST alanine aminotransferase; GGT gamma glutamate transferase; TBIL total bilirubin; DBIL direct bilirubin; IBIL indirect bilirubin. *p<0.05.

### The Significance of the Absence of Rash, Oral Changes and Cervical Lymphadenopathy in Predicting CAA

**FIGURE 1:**
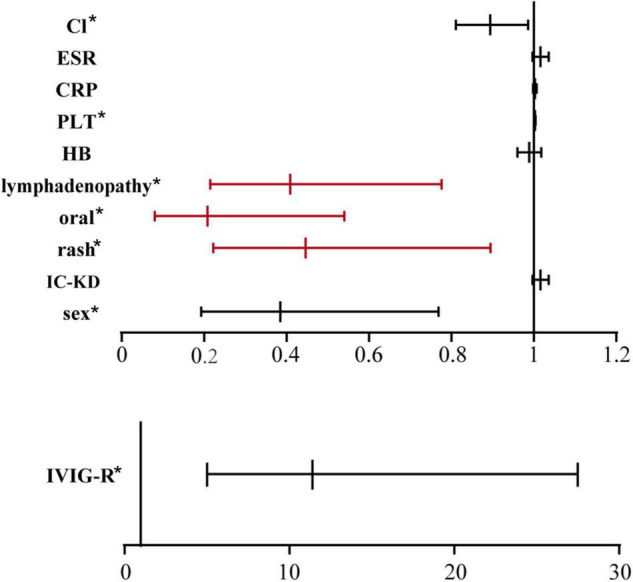
Multivariable-adjusted forest plot for the risks of CAA. IVIG-R was separated from other indices because of the great difference in the abscissa scale. *p < 0.05.

To better identify the risk factors for CAA, logistic regression analysis was further carried out. The results are shown in Table 2. It revealed that sex (female to male) (OR:0.385, 95%CI:0.193, 0.769), rash (OR:0.446, 95%CI:0.222, 0.895), oral changes (OR:0.208, 95%CI:0.08, 0.540), cervical lymphadenopathy (OR:0.409, 95%CI:0.215, 0.776), IVIG-resistance (OR:11.154, 95%CI:4.594, 27.08), PLT (OR:1.003, 95%CI:1.001, 1.004) and Cl levels (OR:0.894, 95%CI:0.811, 0.986) were all relative factors for CAA, which means that the male patients, patients without rash, oral changes, or cervical lymphadenopathy, patients with IVIG-resistance, high PLT levels, and low Cl levels were at higher risk for developing CAA. The results are shown in the form of a forest map in Figure 1, and because of the difference in scale, we divided the forest map into two parts.

**TABLE 2:** Multivariate logistic regression according to CAA.

	OR	95%CI	*P*
Sex	0.385	(0.193, 0.769)	0.007*
Rash	0.446	(0.222, 0.895)	0.023*
Oral	0.208	(0.08, 0.540)	0.001*
Lymphadenopathy	0.409	(0.215, 0.776)	0.006*
IC-KD	1.016	(0.997, 1.036)	0.091
IVIG-R	11.154	(4.594, 27.08)	<0.001*
HB	0.989	(0.960, 1.018)	0.439
PLT	1.003	(1.001, 1.004)	0.007*
CRP	1.003	(0.998, 1.007)	0.233
ESR	1.016	(0.997, 1.036)	0.091
Cl	0.894	(0.811, 0.986)	0.025*

IC-KD, incomplete Kawasaki disease; IVIG-R, intravenous immunoglobulin-resistance; HB, hemoglobin; PLT, platelets; CRP, C-reactive protein; ESR, erythrocyte sedimentation rate; Cl, chlorine; *p<0.05.

**FIGURE 2:**
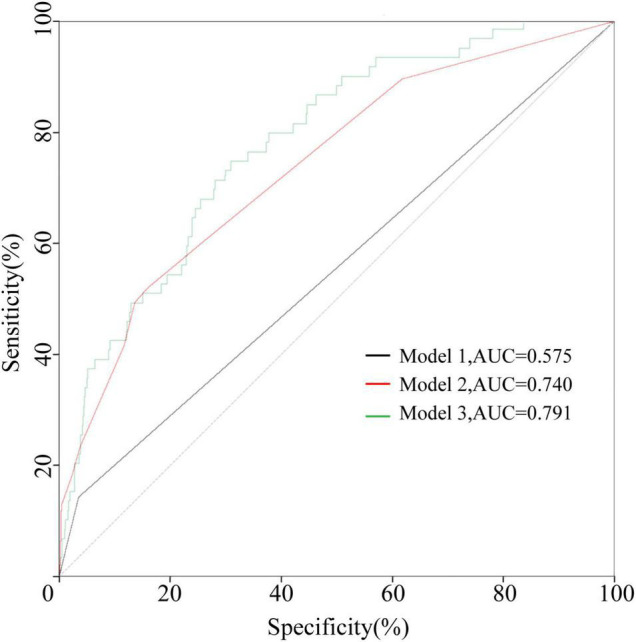
The ROC curves of the three models in the development cohort. Model 1 contained IVIG resistance. Model 2 contained IVIG resistance, rash, oral changes, and cervical lymphadenopathy. Model 3 contained IVIG resistance, rash, oral changes, cervical lymphadenopathy, sex, PLT, and Cl levels.

Combined with the results of our statistical analysis and considering the important role of IVIG resistance in predicting CAA, which has been generally accepted by researchers and proven in our study, three models were established. Model 1 contained only IVIG resistance. Model 2 added three indicators of clinical symptoms (rash, oral changes, and cervical lymphadenopathy). In Model 3, sex and PLT and Cl levels were also added. The specific *p* values and OR values are shown in Table 3. The ROC curves of the three models are plotted in Figure 2. The AUC of Model 3 (AUC = 0.791) was the best, but it was also the most complex model. The AUC of Model 2 (AUC = 0.740) was slightly lower than that of Model 3, but it was simpler and neater than Model 3. Both models were better than Model 1 (AUC = 0.575). Therefore, Model 2 was chosen for further study.

**TABLE 3:** Logistic regression analysis of three models for better predicting CAA.

	Model 1				Model 2				Model 3		
	OR	95%CI	*P*		OR	95%CI	*P*		OR	95%CI	*P*
IVIG-R	4.605	(2.251, 9.420)	<0.001*	IVIG-R	8.195	(3.789, 17.722)	<0.001*	IVIG-R	9.346	(4.128, 21.159)	<0.001*
				Rash	0.307	(0.183, 0.514)	<0.001*	Rash	0.367	(0.213, 0.632)	<0.001*
				Oral	0.158	(0.081, 0.306)	<0.001*	Oral	0.166	(0.081, 0.340)	<0.001*
				Lymphadenopathy	0.387	(0.233, 0.645)	<0.001*	Lymphadenopathy	0.391	(0.229, 0.670)	0.001*
								Sex	0.434	(0.240, 0.785)	0.006*
								PLT	1.003	(1.001, 1.005)	<0.001*
								Cl	0.896	(0.826, 0.973)	0.009*

Model 1 contained IVIG-resistance. Model 2 contained IVIG-resistance, rash, oral changes and cervical lymphadenopathy. Model 3 contained IVIG-resistance, rash, oral changes, cervical lymphadenopathy, sex, PLT and Cl levels, *p<0.05.

### The Significance of the Absence of Rash, Oral Changes and Cervical Lymphadenopathy in Predicting CAA Was Confirmed in the Validation Cohort

**FIGURE 3:**
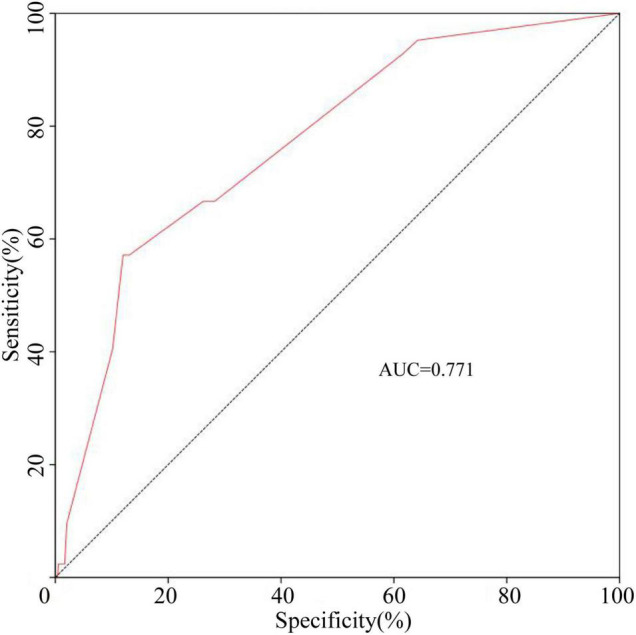
The ROC curve of Model 2 in the validation cohort. Model 2 contained IVIG resistance, rash, oral changes, and cervical lymphadenopathy.

**FIGURE 4:**
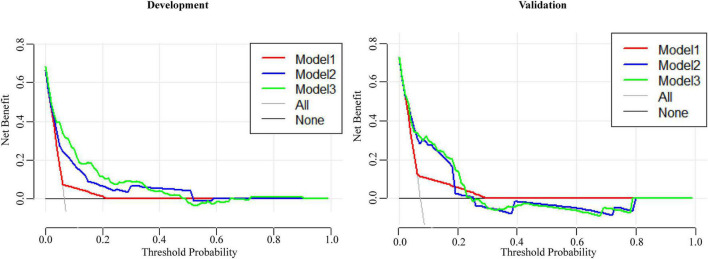
The DCAs of three models for predicting CAA in the development cohort and the validation cohort.

The ROC curve of Model 2 was carried out on the validation cohort, and the AUC of Model 2 in the validation cohort was greater than 0.7 (Figure 3). To study further, the DCAs for the development cohort and the validation cohort of the three models were performed, and are shown in Figure 4. There was no significant difference between Model 2 and Model 3, and both Model 2 and Model 3 were superior to Model 1; thus, we discovered the importance of the absence of three clinical symptoms (rash, oral changes, and cervical lymphadenopathy) in addition to IVIG resistance in predicting CAA.

### The Absence of Rash, Oral Changes, and Cervical Lymphadenopathy Were Better Predictors of CAA in Female Patients

**FIGURE 5:**
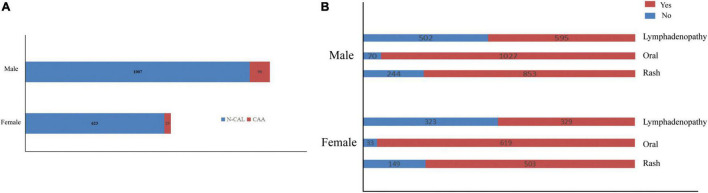
Bar charts of the total cohort classified by sex were drawn to describe **(A)** the distributions of CAA and **(B)** the symptoms of rash, oral changes, and cervical lymphadenopathy.

**FIGURE 6:**
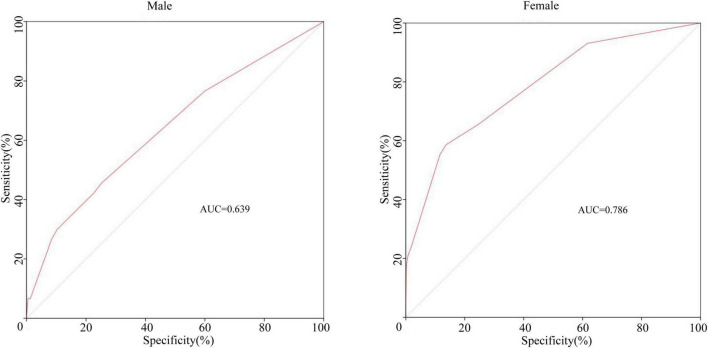
The ROC curves of the combining symptoms (rash, oral changes, and cervical lymphadenopathy) classified by sex for predicting CAA.

Previous studies have suggested the importance of sex in predicting CAA. Considering this, we grouped the total cohort according to sex and CAA status and focused on the distribution of rash, oral changes, and cervical lymphadenopathy between groups. The description is shown in Figure 5. We compared the items enrolled in the three models above between the N-CAL and CAA groups in different sexes, and the results are shown in Table 4. The results show that the symptoms of rash, oral changes, and cervical lymphadenopathy, which we were interested in, all had statistically significant differences between N-CAL and CAA patients in both male and female patients. Then, we compared the three symptoms through the ROC curves in predicting CAA with different genders and found an interesting phenomenon. In female patients with KD, the absence of rash, oral changes, and cervical lymphadenopathy had a greater effectiveness in predicting CAA than in male patients, which was demonstrated by the superior AUC in females (AUC = 0.786) than in males (AUC = 0.639). The specific *p* values are displayed in Table 5, and the ROC curves are shown in Figure 6.

**TABLE 4:** The comparison between N-CAL and CAA groups in male and female patients.

	Male			Female		
	N-CAL	CAA	*P*	N-CAL	CAA	*P*
Rash	798 (79.2%)	55 (61.1%)	<0.001*	491 (78.8%)	12 (41.4%)	<0.001*
Oral	949 (94.2%)	78 (86.7%)	0.011*	598 (96%)	21 (72.4%)	<0.001*
Lymphadenopathy	560 (55.6%)	35 (38.9)	0.003*	323 (51.8%)	6 (20.7%)	0.001*
IVIG-R	38 (3.8%)	17 (18.9%)	<0.001*	19 (3%)	2 (6.9%)	0.239
PLT	365.09 ± 125.72	415.05 ± 156.87	0.005*	374.34 ± 126.45	462.44 ± 205.63	0.016*
Cl	101.81 ± 3.09	100.94 ± 2.74	0.005*	101.60 ± 3.03	102.18 ± 3.39	0.461

IVIG-R, intravenous immunoglobulin-resistance; PLT, platelets; Cl, chlorine; *p<0.05.

**TABLE 5:** Logistic regression analysis of rash, oral and lymphadenopathy in male and female patients.

	Male			Female		
	OR	95%CI	*P*	OR	95%CI	*P*
Rash	0.399	(0.253, 0.631)	<0.001*	0.19	(0.085, 0.426)	<0.001*
Oral	0.42	(0.213, 0.829)	0.012*	0.096	(0.035, 0.266)	<0.001*
Lymphadenopathy	0.485	(0.310, 0.759)	0.002*	0.215	(0.083, 0.561)	0.002*

Logistic regression analysis of rash, oral and lymphadenopathy in male and female patients. *p < 0.05.

## Discussion

KD is an acute systemic vasculitis affecting young children, and its incidence is rising sharply (19–21). The main clinical manifestations are fever, rash, oral changes, anomalies of the extremities, bilateral non-exudative conjunctivitis, and cervical lymphadenopathy. KD also affects other systems and leads to symptoms such as vomiting, aseptic pyuria (22), and even aseptic encephalitis (23).

Coronary artery involvement is a frequent complication of KD. According to statistics, 20–25% of KD patients develop severe complications, such as CAA (24), myocardial infarction, and heart failure (25), and a giant aneurysm can occur particularly in untreated patients during the subacute phase. Studies have shown either persistence or stenosis without regression as a long-term consequence of giant coronary aneurysm (GCA). Only some cases reported a regression of GCA in KD (26, 27).

Researchers have done much to identify the risk factors for CAA. Huang et al. (28) found that CAA was significantly related to younger age of onset, higher platelet count, and lower serum albumin levels. A large CAA in patients with KD was related to lower serum sodium levels. Researchers also explained that low levels of serum albumin and immunoglobulin M were risk factors for CAA (29). In addition, male sex, young age (under 1 year old) and high PLT count were considered to be risk factors for CAA in KD patients by Downie (30).

In our study, we also found that the CAA group had lower hemoglobin, higher platelet count, and elevated CRP levels, and that CAA occurred more commonly in male patients. However, age, serum sodium, and serum albumin were not found to be different between the two groups. After regression statistical analysis, hemoglobin, platelet count, and CRP were not found to be independent risk factors for CAA. According to our statistical analysis, the existence of IVIG resistance and the absence of rash, oral changes, and cervical lymphadenopathy play important roles in the occurrence of CAA events, and this result has been well verified in the validation cohort.

Previous studies have shown that sex plays an important role in the occurrence of CAA (31, 32). Therefore, a separate statistical analysis was carried out on different sexes. The results showed that the absence of the three clinical symptoms (rash, oral changes, and cervical lymphadenopathy) were better predictors for CAA, especially in female patients. The sensitivity of the combination of the three symptoms in predicting CAA was higher than that in male patients. This result has not been reported in other studies.

IVIG resistance has been shown to be the most important risk factor for CAA. Our study indicated that as well. In addition, our study also found that without rash, oral changes, and cervical lymphadenopathy, the risk of CAA was higher than that of patients with the above symptoms, especially in female patients. We found that some of the clinical indicators currently investigated were less sensitive to CAA than the clinical symptoms themselves. This suggested that in addition to searching for biomarkers that can recognize CAA early, we should not forget the importance of recognizing clinical symptoms for the disease. At the same time, the research reminds us to pay more attention to the incidence of CAA in female patients.

The absence of rash, oral changes, and cervical lymphadenopathy at the same time is consistent with the incomplete KD diagnosis. Taking this into account, we included incomplete Kawasaki disease as an index in the statistics. A statistically significant difference was observed between the two groups, but the index was not included after the regression analysis. This may be related to the difficulty in identifying incomplete KD. Although the diagnoses of our patients are all conducted by experienced physicians, with the rapid development of understanding of incomplete KD, older cases may have not recognized this diagnosis. Meanwhile, the number of cases in the CAA group was still not high, and more patients need to be included. Multicenter studies will be helpful. Additionally, long-term complications and the regression of CAA, especially large CAA, should be given more attention.

In conclusion, the absence of rash, oral changes, and cervical lymphadenopathy are risk factors for CAA, especially in female patients. Accurate recognition of symptoms, early diagnosis, and standard treatment for KD are key to reducing the incidence of CAA.

## Data Availability Statement

The raw data supporting the conclusions of this article will be made available by the authors, without undue reservation.

## Ethics Statement

The studies involving human participants were reviewed and approved by the Ethical Decision Committee of the Research Administration at Second Affiliated Hospital and Yuying Children’s Hospital of Wenzhou Medical University. Written informed consent to participate in this study was provided by the participants’ legal guardian/next of kin. Written informed consent was obtained from the individual(s), and minor(s)’ legal guardian/next of kin, for the publication of any potentially identifiable images or data included in this article.

## Author Contributions

HY was responsible for data statistics and writing manuscript. WS was responsible for data collection and manuscript revising. HW was responsible for data collection and part of writing. SZ, ZX, RW, XR, and HQ collected the data. JZ, CZ, and MC provided the resources and designed the study. All authors contributed to the article and approved the submitted version.

## Conflict of Interest

The authors declare that the research was conducted in the absence of any commercial or financial relationships that could be construed as a potential conflict of interest.

## Publisher’s Note

All claims expressed in this article are solely those of the authors and do not necessarily represent those of their affiliated organizations, or those of the publisher, the editors and the reviewers. Any product that may be evaluated in this article, or claim that may be made by its manufacturer, is not guaranteed or endorsed by the publisher.
